# Factores relacionados con la mortalidad en pacientes con enfermedad pulmonar obstructiva crónica en población colombiana

**DOI:** 10.7705/biomedica.7140

**Published:** 2024-05-31

**Authors:** Eduardo Tuta-Quintero, Alirio R. Bastidas, Luis F. Giraldo-Cadavid, Juliana Echeverri, Juan D. Botero, Valentina Villarreal, Camila Zambrano, Valeria Rabe, Juan Hernández, Daniel Tavera, Juan Acosta, Ángela Martínez, Carlos Granados, María Nieto, Sergio E. Román, William A. Achury, Jonathan Guezguan-Pérez, Paula Prieto, Diana Parra-Cárdenas

**Affiliations:** 1 Facultad de Medicina, Universidad de La Sabana, Chía, Colombia Universidad de La Sabana Facultad de Medicina Universidad de La Sabana Chía Colombia; 2 Departamento de Neumología Intervencionista, Fundación Neumológica Colombiana, Bogotá, D.C., Colombia Fundación Neumológica Colombiana Departamento de Neumología Intervencionista Fundación Neumológica Colombiana Bogotá, D.C. Colombia

**Keywords:** enfermedad pulmonar obstructiva crónica, mortalidad, factores de riesgo, estudio observacional, Pulmonary disease, chronic obstructive, mortality, risk factors, observational study

## Abstract

**Introducción.:**

En los países de medianos y bajos ingresos, los datos sobre la mortalidad y los factores de riesgo en pacientes con enfermedad pulmonar obstructiva crónica son limitados.

**Objetivo.:**

Identificar la incidencia de muerte y sus variables relacionadas en una población colombiana durante 12 meses de seguimiento.

**Materiales y métodos.:**

Se llevó a cabo un estudio retrospectivo de sujetos con diagnóstico de enfermedad pulmonar obstructiva crónica en una clínica de tercer nivel en Colombia. Los cocientes de probabilidades se calcularon mediante un análisis de regresión logística multivariable con la variable de resultado “mortalidad a los 12 meses”.

**Resultados.:**

Ingresaron 524 pacientes, de los cuales el 18,1 % (95 / 524) murió. La edad promedio fue de 69,7 (DE = 8,92) y el 59,2 % (310 / 524) eran mujeres. Las variables asociadas con la mortalidad fueron la edad (OR = 6,54; IC_95%_: 3,65-11,36; p < 0,001), años de exposición al humo de leña (OR = 4,59; IC_95%_: 1,64-12,82; p = 0,002), insuficiencia cardiaca crónica (OR = 1,81; IC_95%_: 1,13-2,91; p = 0,014), enfermedad cerebrovascular (OR = 3,35; IC_95%_: 1,04-10,75; p = 0,032) y enfermedad renal crónica (OR=6,96; IC _95%_:1,15- 41,67; p = 0,015). Al ajustar las variables en el análisis multivariado únicamente se mostró asociación entre el sexo (OR = 1,55; IC_95%_: 0,95-2,54; p = 0,008) y la edad (OR = 5,94; IC_95%_: 3,3-10,69; p < 0,001).

**Conclusión.:**

La edad, los años de exposición al humo de leña, la insuficiencia cardiaca crónica, la enfermedad cerebrovascular y la renal crónica fueron variables clínicas asociadas a un desenlace fatal. Sin embargo, la edad y el sexo fueron las únicas relacionadas con la mortalidad al ajustarlas por factores de confusión.

## Abstract (en)

**Introduction.:**

Data in low- and middle-income countries on mortality and its related risk factors in patients with chronic obstructive pulmonary disease are limited.

**Objective.:**

To identify the incidence of death and its relationship with variables in a Colombian population during 12 months of follow-up.

**Materials and methods.:**

We carried out a retrospective study in subjects diagnosed with chronic obstructive pulmonary disease in a third-level hospital in Colombia. Odds ratios were calculated using multivariable logistic regression analysis with the outcome variable “mortality at 12 months”.

**Results.:**

We included 524 patients, 18.1% (95 / 524) died. The average age was 69.7 (SD = 8.92), and 59.2% (310 / 524) were women. The variables associated with mortality were age (OR = 6.54; 95% CI = 3.65-11.36; p < 0.001), years of exposure to wood smoke (OR = 4.59; 95% CI = 1.64-2.82; p = 0.002), chronic heart failure (OR = 1.81; 95% CI = 1.132.91; p = 0.014), cerebrovascular disease (OR = 3.35; 95% CI = 1.04-10.75; p = 0.032), and chronic kidney disease (OR = 6.96; 95% CI = 1.15-41.67; p=0.015). When adjusting the variables in the multivariate analysis, only an association was found for sex (OR = 1.55; 95% CI = 0.95-2.54; p = 0.008) and age (OR = 5.94; 95% CI = 3.3-10.69; p < 0.001).

**Conclusion.:**

Age, years of exposure to wood smoke, chronic heart failure, and cerebrovascular and chronic kidney disease were the clinical variables associated with a fatal outcome. However, age and sex were the only variables related to mortality when adjusted for confounding factors.

La enfermedad pulmonar obstructiva crónica (EPOC) es una condición heterogénea con síntomas respiratorios como disnea, tos, expectoración o exacerbaciones debido a una obstrucción persistente y, a menudo, progresiva del flujo de aire ^1^^,^^2^. La prevalencia de la EPOC se calcula alrededor del 10,3 % (IC_95%_: 8,2-12,8) y se estima que más de 545 millones de personas presentan esta enfermedad ^3^^,^^4^. Los principales factores de riesgo de la EPOC son la exposición persistente a contaminantes ambientales y el envejecimiento de la población ^3^^,^^4^. Esta enfermedad ocupa la tercera causa de morbimortalidad a nivel mundial y representa la quinta carga económica, con un costo anual promedio de USD$ 100.000 millones ^1^^,^^2^. La EPOC es responsable, aproximadamente, de 41,9 muertes por cada 100.000 personas y muchos pacientes con esta condición están subdiagnosticados o reciben terapias subóptimas ^1^^-^^3^.

Una actualización sobre la carga de morbimortalidad asociada a la EPOC revela cambios significativos en las tasas de mortalidad entre 2005-2007 y 2015-2017. Durante este período, la mortalidad global disminuyó 16 % en los hombres, con tasas anuales de 14,0 por cada 100.000, mientras que, en las mujeres, se observó un aumento del 13 %, con tasas anuales de 6,4 por cada 100.000 ^5^. En Europa, se registraron tasas de mortalidad estandarizadas por edad de 3.230 por cada 100.000 hombres y 2.202 por cada 100.000 mujeres para el año 2019 ^6^. Los estudios poblacionales de países latinoamericanos, asiáticos y de Oceanía han descrito un aumento en la mortalidad de las mujeres, incluso alcanzando incidencias mayores a las descritas en hombres ^5^^-^^7^.

Dentro del espectro de enfermedades respiratorias crónicas, la EPOC mantiene su posición como la más prevalente y la que presenta una tasa de mortalidad más elevada ^8^^,^^9^. Su pronóstico se encuentra intrínsecamente vinculado a diversos factores, como la gravedad de la obstrucción del flujo de aire, la frecuencia de exacerbaciones, la edad (especialmente en individuos mayores de 60 años) y la presencia de comorbilidades como hipertensión arterial sistémica, cáncer pulmonar, insuficiencia cardiaca crónica y osteoporosis, entre otras ^9^. Las comorbilidades suelen ser una constante en pacientes diagnosticados con EPOC y tienen el potencial de agravar la evolución clínica de los síntomas respiratorios, las exacerbaciones y las hospitalizaciones ^10^. Esto, a su vez, se traduce en un aumento directo de las tasas de mortalidad, el número de hospitalizaciones y los costos asociados al tratamiento ^10^^,^^11^.

Dada la repercusión funcional atribuida a la prevalencia elevada de comorbilidades, el deterioro físico y la fragilidad que caracterizan a los pacientes con EPOC, se plantea la posibilidad de que diversos factores de riesgo estén vinculados con la mortalidad ^8^^,^^10^^,^^11^. No obstante, los estudios disponibles han arrojado resultados ambiguos al respecto, especialmente en los países de ingresos bajos y medios donde la enfermedad es una carga considerable para el sistema de salud ^8^^,^^11^.

El objetivo de este estudio fue identificar la incidencia de muerte y las diferentes variables que pueden estar asociadas con un desenlace fatal en pacientes con EPOC en una población colombiana durante 12 meses de seguimiento.

## Materiales y métodos

Se realizó un estudio de cohorte, retrospectivo, en sujetos que recibieron atención médica y se sometieron a pruebas de función pulmonar en una clínica de tercer nivel en Colombia durante el período entre el 2015 y el 2019. La revisión de los registros clínicos se efectuó entre los años 2020 y 2021.

### Criterios de selección

Se incluyeron pacientes mayores de 40 años que habían sido diagnosticados con EPOC. Este diagnóstico se basó en la presencia de síntomas característicos, como disnea, tos, expectoración o sibilancias, además de exposición previa a factores de riesgo asociados con la enfermedad. Además, se evaluó la obstrucción del flujo de aire mediante la medición de la relación de la capacidad vital forzada y el volumen espiratorio forzado en el primer segundo (VEF_1_ / CVF), después de la administración de un broncodilatador durante la espirometría, y se consideró obstructivo cuando esta relación fue menor de 0,7 o menor del límite inferior de lo normal ^4^^,^^11^. Se excluyeron pacientes sin información clínica o supervivencia a los 12 meses de seguimiento. Una vez encontrada una historia clínica que cumplía con los criterios de inclusión, se revisaron los desenlaces de interés registrados en los siguientes doce meses.

### Variables

La variable dependiente fue la mortalidad a 12 meses y como variables independientes se incluyeron edad, sexo, antecedentes de comorbilidades, características durante el examen físico, exámenes de laboratorio y variables de la espirometría como la capacidad vital forzada (CVF), volumen espiratorio forzado en el primer segundo (FEV_1_) y relación VEF_1_ / CVF.

### Tamaño de muestra

Se ingresaron todos los sujetos que cumplían los criterios de selección del período de estudio. La información se ingresó de manera consecutiva y la recolección de datos fue realizada por personas del grupo de investigación con entrenamiento previo sobre el protocolo y la información por recolectar; se hizo un proceso de doble verificación al momento de transcribir los datos a la base electrónica para disminuir las probabilidades de sesgo de transcripción.

### Análisis estadístico

Los datos fueron recopilados en el software de captura de datos electrónicos *Research Electronic Data Capture* (REDCap) y, posteriormente, analizados en el programa SPSS™, versión 25. Las variables cuantitativas se resumieron en promedios y desviaciones estándar si la distribución era normal, y mediana y rango intercuartílico si la distribución no era normal; las variables cualitativas se resumieron en frecuencias y porcentajes.

La incidencia de mortalidad se obtuvo dividiendo el total de muertes sobre la población de pacientes con diagnóstico de EPOC. Se realizó un análisis bivariado comparando variables cuantitativas de distribución normal con la prueba t de Student y variables de distribución no normal con la prueba de U Mann-Whitney; las variables cualitativas se compararon mediante la prueba exacta de Fisher. Las razones de probabilidad (*odds ratio*, OR) se calcularon inicialmente para todas las variables y luego se ajustaron mediante una regresión logística binaria con las variables que presentaran plausibilidad biológica para el desenlace de mortalidad y significancia estadística en el análisis bivariado inicial. Se aplicó imputación múltiple para los valores faltantes durante el seguimiento, con un máximo de 9,5 %. Se consideró como estadísticamente significativo un valor p inferior a 0,05.

### Consideraciones éticas

El presente trabajo cumplió con las normas éticas contempladas en la Declaración de Helsinki y fue aprobado por el Comité de Ética de la Universidad de La Sabana. Se consideró como una investigación sin riesgo según las recomendaciones de la Resolución 8430 de 1993 para investigación en seres humanos. La información recolectada fue manejada de acuerdo con la ley Habeas Data para la protección de datos personales, vigente para Colombia.

## Resultados

### Características clínicas

**Figura 1.**
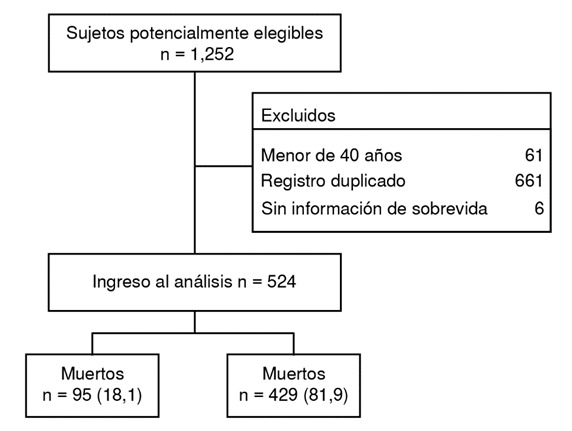
Flujo de ingreso de sujetos al estudio

Se incluyeron 524 pacientes, el 18,1 % (95 / 524) con desenlace fatal (figura 1). La edad promedio de la población fue de 69,7 (DE = 8,92) y el 59,2 % (310 / 524) eran mujeres. La cardiopatía isquémica (27,4 % versus 26,1 %; p = 0,801) y la insuficiencia cardiaca crónica (35,8 % *vs.* 23,5 %; p = 0,014) se presentaron con mayor frecuencia en los pacientes que murieron respecto al grupo control (cuadro 1).

**Cuadro 1.** Características generales de la población (N = 524)

	Muertos (n = 95)	Vivos (n = 429)	p	Total
	[media ± DE]	[media ± DE]		[media ± DE]
Mujer	50 ± 52,6)	260 ± 60,6)	0,152	310 ± 59,2)
Edad (años)	80,8 ± 11,76)	67,2 ± 12,96)	< 0,001	69,7 ± 13,77)
Peso (kg)	82,2 ± 6,28)	79,5 ± 9,34)	0,005	80,1 ± 8,92)
Talla (m)	1,70 ± 0,08)	1,69 ± 0,09)	0,167	1,61 ± 0,09)
Índice de masa corporal (kg/m2)	30,1 ± 3,74)	29,7 ± 4,5)	0,426	29,8 ± 4,37)
PAS mm Hg	128,2 ± 15,88)	126,3 ± 16,83)	0,406	126,6 ± 16,66)
PAD mm Hg	76,7 ± 11,51)	75,5 ± 10,31)	0,398	75,7 ± 10,53)
FC (latidos)	74,3 ± 9,01)	76,8 ± 10,71)	0,057	76,3 ± 10,46)
FR (respiraciones por minuto)	18,4 ± 2,15)	18,4 ± 2,02)	0,982	18,4 ± 2,04)
Saturación (%)	92,2 ± 3,49)	92 ± 3,81)	0,686	92,1 ± 3,75)
Temperatura (° C)	36,1 ± 0,29)	36,2 ± 0,35)	0,456	36,2 ± 0,34)
	**n (%)**	**n (%)**		**n (%)**
¿Ha fumado cigarrillo?	44 (46,3)	202 (47,1)	0,892	246 (46,9)
Índice de paquetes al año [media ± DE]	16,3 ± 19,6)	14,2 ± 18,84)	0,427	14,6 ± 18,97)
¿Expuesto al humo de leña?	34 (35,8)	119 (27,7)	0,118	153 ± 29,2)
Años expuesto al humo de leña [media ± DE]	19,1 ± 7,8)	21,5 ± 9,42)	0,029	21 ± 9,12)
	**n (%)**	**n (%)**		**n (%)**
Infarto agudo de miocardio	26 (27,4)	112 (26,1)	0,801	138 (26,3)
Insuficiencia cardiaca crónica	34 (35,8)	101 (23,5)	0,014	135 (25,8)
Enfermedad cerebrovascular	5 (5,3)	7 (1,6)	0,032	12 (2,3)
Enfermedad del tejido conectivo	3 (3,2)	3 (0,7)	0,042	6 (1,1)
Úlcera péptica	2 (2,1)	2 (0,5)	0,097	4 (0,8)
Diabetes	2 (2,1)	2 (0,5)	0,097	4 (0,8)
Enfermedad renal crónica	3 (3,2)	2 (0,5)	0,015	5 (1)
Linfoma-mieloma múltiple	1 (1,1)	(0)	0,054	1 (0,2)
Tumor sólido no metastásico	4 (4,2)	(0)	< 0,001	4 (0,8)
Tumor sólido metastásico	1 (1,1)	(0)	0,054	1 (0,2)

PAS: presión arterial sistólica; PAD: presión arterial diastólica; FC: frecuencia cardiaca; FR: frecuencia respiratoria; DE: desviación estándar

### Pruebas de función pulmonar y exámenes de laboratorio

La relación VEF_1_ / CVF antes del broncodilatador B2 fue de 73,9 (DE = 13,49) en los pacientes con desenlace fatal frente a 74,5 (DE = 12,41) en aquellos con desenlace diferente (p = 0,978) (cuadro 2), mientras que después del broncodilatador fue de 76,6 (DE = 12,99) en los pacientes con desenlace fatal frente a 76,4 (DE = 11,65) en los demás (p = 0,866). La proteína C reactiva fue 43,1 mg/L (DE = 68,75) en el grupo de pacientes que fallecieron frente a 28,6 mg/L (DE = 62,79) (p = 0,095) en aquellos que no.

**Cuadro 2.** Pruebas de función pulmonar y exámenes de laboratorio (N = 524)

Variables	Muertos (n = 95) media ± DE	Vivos (n = 429) media ± DE	p	Total media ± DE
CVF pre B_2_ (L)	3,50 ± 0,82	3,63 ± 0,85	0,426	3,61 ± 0,85
CVF pre B_2_ %	95,7 ± 17,54	97,0 ± 15,87	0,569	96,8 ± 16,17
CVF post B_2_ (L)	3,63 ± 0,74	3,76 ± 0,77	0,371	3,71 ± 0,77
CVF post B_2_ %	98,5 ± 16,85	99,1 ± 16,82	0,797	99,0 ± 16,82
VEF_1_ pre B_2_ (L)	2,61 ± 0,72	2,78 ± 0,75	0,350	2,68 ± 0,75
VEF_1_ pre B_2_ %	89,2 ± 21,27	91,6 ± 20,69	0,393	91,2 ± 20,8
VEF_1_ post B_2_ (L)	2,71 ± 0,67	2,89 ± 0,7	0,294	2,80 ± 0,7
VEF_1_ post B_2_ %	97,30 ± 20,64	97,37 ± 18,28	0,988	97,31 ± 18,71
VEF_1_ / CVF pre B_2_	73,9 ± 13,49	74,5 ± 12,41	0,978	74,1 ± 12,6
VEF_1_ / CVF pre B_2_ %	94,2 ± 16,87	94,9 ± 15,05	0,750	94,7 ± 15,38
VEF_1_ / CVF post B_2_	76,6 ± 12,99	76,4 ± 11,65	0,866	76,4 ± 11,89
VEF_1_ / CVF post B_2_ %	91,0 ± 10,73	89,1 ± 11,01	0,200	89,5 ± 10,98
Poblaciones celulares (células/ml)				
Glóbulos blancos	7.736,1 ± 2.362,09	7.762,9 ± 2.366,61	0,933	7.758,1 ± 2.363,56
Neutrófilos	4.726,8 ± 2.433,59	4.742,3 ± 2.463,59	0,963	4.739,5 ± 2.455,87
Linfocitos	2.163,7 ± 847,01	2.073,2 ± 796,95	0,406	2.089,4 ± 806,18
Monocitos	617,7 ± 187,59	621,7 ± 192,43	0,877	621,0 ± 191,39
Eosinófilos	224,1 ± 209,85	219,5 ± 220,11	0,877	220,4 ± 218,1
Basófilos	40,4 ± 45,53	43,9 ± 61,94	0,597	43,5 ± 59,35
Glóbulos rojos	5,12 ± 0,47	5,13 ± 0,47	0,935	5,10 ± 0,47
Plaquetas	252.210,5 ± 77.644,43	261.424,2 ± 81.726,25	0,399 259.753,8 ± 81.006,87	
Hemoglobina (g/dl)	15,4 ± 1,46	15,3 ± 1,4	0,734	15,3 ± 1,41
Hematocrito (%)	45,1 ± 3,59	45,3 ± 3,51	0,800	45,2 ± 3,52
Proteína C reactiva (mg/L)	43,1 ± 68,75	28,6 ± 62,79	0,095	31,8 ± 64,17
pH	7,40 ± 0,03	7,45 ± 0,03	0,867	7,41 ± 0,03
PaO2 (mm Hg)	59,61 ± 9,62	59,71 ± 9,4	0,974	59,72 ± 9,43
PaCO2 (mm Hg)	35,3 ± 6,8	35,1 ± 5,92	0,890	35,2 ± 6,09
HCO3 (mEq/L)	22,5 ± 4,47	22,3 ± 3,88	0,722	22,3 ± 3,99
Base exceso (mEq/L)	-0,8 ± 3,73	-1,1 ± 3,3	0,483	-1,1 ± 3,38
PaO2 / FiO2	274,1 ± 51,4	271,8 ± 45,1	0,726	272,2 ± 46,26
FiO2	21,7 ± 1,92	21,9 ± 2,36	0,399	21,9 ± 2,29

CVF: capacidad vital forzada; B2: broncodilatador;VEF_1_: volumen espiratorio forzado en el primer segundo; VEF1 / CVF: relación capacidad vital forzada / volumen espiratorio forzado en el primer segundo

### Vacunas

La proporción de pacientes vacunados contra la influenza fue del 9,2 % (26,3 % en pacientes fallecidos versus 17,1 % en sobrevivientes; p = 0,055) y de usuarios de anticolinérgicos de corta y larga acción fue del 4,9 % (6,3 % en fallecidos versus 1,4 % en sobrevivientes; p = 0,004). Las características de los tratamientos utilizados en la población se muestran en el cuadro 3.

**Cuadro 3.** Tratamientos (N = 524)

	Muertos (n = 95)	Vivos (n = 429)	p	Total n (%)
	n (%)	n (%)		
Vacuna influenza	25 (26,3)	76 (17,7)	0,055	101 (19,3)
Vacuna neumococo	2 (2,1)	8 (1,9)	0,871	10 (1,9)
SABA	18 (18,9)	78 (18,2)	0,861	96 (18,3)
LABA	2 (2,1)	4 (0,9)	0,331	6 (1,1)
SAMA	9 (9,5)	55 (12,8)	0,367	64 (12,2)
LAMA	5 (5,3)	25 (5,8)	0,830	30 (5,7)
SABA + LAMA	1 (1,1)	2 (0,5)	0,493	3 (0,6)
LABA + LAMA	6 (6,3)	6 (1,4)	0,004	12 (2,3)
Metilxantinas	1 (1,1)	11 (2,6)	0,004	12 (2,3)
LABA + corticoide	5 (5,3)	44 (10,3)	0,130	49 (9,4)
LABA + LAMA + corticoide	(0)	2 (0,5)	0,775	2 (0,4)
Roflumilast	(0)	1 (0,2)	0,840	1 (0,2)
Mucolítico	1 (1,1)	2 (0,5)	0,493	3 (0,6)
Corticoide inhalado	11 (11,6)	39 (9,1)	0,455	50 (9,5)
Corticoide vía oral	2 (2,1)	9 (2,1)	0,996	11 (2,1)
Antibiótico	(0)	3 (0,7)	0,724	3 (0,6)
Oxígeno	13 (13,7)	56 (13,1)	0,869	69 (13,2)
Oxígeno > 16 horas	11 (11,6)	44 (10,3)	0,704	55 (10,5)
Oxígeno < 16 horas	4 (4,2)	29 (6,8)	0,355	33 (6,3)

SABA: *short-acting @2 agonist;* LABA: *long-acting @2 agonist;* SAMA: *short-acting muscarinic antagonist*; LAMA: *long-acting muscarinic antagonist*

### Factores de riesgo asociados a la mortalidad

Las variables asociadas con la mortalidad fueron la edad (OR = 6,54; IC_95%_: 3,65-11,36; p < 0,001), los años de exposición al humo de leña (OR = 4,59; IC_95%_: 1,64-12,82; p = 0,002), insuficiencia cardiaca crónica (OR = 1,81; IC_95%_: 1,13-2,91; p = 0,014), enfermedad cerebrovascular (OR = 3,35; IC_95%_: 1,04-10,75; p = 0,032) y enfermedad renal crónica (OR = 6,96; IC_95%_: 1,15-41,67; p = 0,015). Al ajustar las variables en el análisis multivariado, únicamente se encontró asociación con el sexo (OR = 1,55; IC_95%_: 0,95-2,54; p = 0,008) y la edad (OR = 5,94; IC_95%_: 3,3-10,69; p < 0,001). Los riesgos relativos se muestran en el cuadro 4.

**Cuadro 4.** Asociación entre las variables con la mortalidad

	OR (IC_95_ %)	p	OR^*^ (^IC^_95_ %*)*	p	RR
Sexo	1,38 (0,89-2,16)	0,152	1,55 (0,95-2,54)	0,008	1,30
Edad	6,45 (3,65-11,36)	< 0,001	5,94 (3,3-10,69)	< 0,001	4,83
Índice de paquetes al año > 20	1,12 (0,58-2,16)	0,747	-	-	4,83
Años expuesto a humo de leña	4,59 (1,64-12,82)	0,002 -	-	-	3,44
Infarto agudo de miocardio	1,07 (0,65-1,76)	0,801	0,72 (0,35-1,46)	0,355	1,05
Insuficiencia cardiaca crónica	1,81 (1,13-2,91)	0,014	1,74 (0,87-3,51)	0,120	1,61
Enfermedad vascular periférica	1,21 (0,78-1,9)	0,391	1,03 (0,63-1,69)	0,910	1,17
Enfermedad cerebrovascular	3,35 (1,04-10,75)	0,032	1,82 (0,41-8,10)	0,433	3,35
Demencia	3,05 (0,5-18,52)	0,202	1,02 (0,11-9,29)	0,988	2,23
Enfermedad del tejido conectivo	4,63 (0,92-23,26)	0,042	3,01 (0,49-18,45)	0,234	2,82
Úlcera péptica	4,59 (0,64-33,33)	0,097	1,59 (0,19-13,38)	0,669	2,80
Diabetes mellitus	4,59 (0,64-33,33)	0,097	1,73 (0,18-16,48)	0,632	2,80
Enfermedad renal crónica	6,96 (1,15-41,67)	0,015	2,74 (0,38-19,82)	0,317	3,38
Proteína C reactiva (mg/l > 20)	2,41 (0,94-6,21)	0,063	-	-	1,90
Eosinófilos (células/ml > 300	1,03 (0,63-1,69)	0,893	0,98 (0,57-1,66)	0,927	1,03
Hiperinsuflación pulmonar	3,46 (0,76-15,63)	0,088	4,44 (0,51-38,79)	0,178	2,40
Enfisema pulmonar	1,99 (0,75-5,32)	0,162	1,04 (0,24-4,52)	0,961	1,70

OR: *odds ratio*; RR: riesgo relativo

* *Odds ratio* ajustado

## Discusión

En este estudio se encontró que los factores asociados con la mortalidad de pacientes diagnosticados con EPOC, durante un período de seguimiento de 12 meses, fueron edad, peso, años de exposición al humo de leña, insuficiencia cardiaca crónica y enfermedades de tipo cerebrovascular, renal crónica, del tejido conjuntivo y tumoral. Sin embargo, la edad y el sexo fueron las únicas variables relacionadas con la mortalidad en el análisis multivariado ajustado por factores de confusión. Los pacientes que murieron mostraron un mayor uso de anticolinérgicos y vacunas.

Ellingsen y colaboradores analizaron factores de riesgo asociados con la mortalidad en 17.745 pacientes diagnosticados con EPOC, de los cuales falleció el 32,5 % ^12^. Las comorbilidades asociadas con la mortalidad fueron insuficiencia cardiaca crónica (*hazard ratio,* HR = 1,88; IC_95%_: 1,74 - 2,04), enfermedad cerebrovascular (HR = 1,52; IC_95%_: 1,40 - 1,64) e infarto agudo de miocardio (HR = 1,40; IC_95%_: 1,24 - 1,58). Giezeman y colaboradores demostraron que la cardiopatía isquémica o insuficiencia cardíaca crónica se asociaba con mayor riesgo de hospitalización y mortalidad por cualquier causa (HR = 1,55; IC_95%_: 1,32 - 1,82 en pacientes con enfermedad pulmonar crónica ^13^.

Los datos del presente estudio y los descritos por Ellingsen y Giezeman concuerdan en la asociación de las enfermedades cardiovasculares, como la insuficiencia cardiaca crónica, el accidente cerebrovascular y el infarto agudo de miocardio, y un desenlace fatal en la población con EPOC (1214). A diferencia de lo reportado en la actualidad, este trabajo señala que la enfermedad renal crónica y la exposición al humo de leña podrían estar vinculados con la mortalidad, lo cual posiblemente esté relacionado con la población de estudio, que es más longeva y con múltiples comorbilidades.

Varios estudios han destacado que en la población diagnosticada con EPOC los hombres de edad avanzada presentan una notable tasa de mortalidad. Esto se confirma como un factor de riesgo significativo para morir cuando el modelo se ajusta mediante análisis multivariados, hallazgos que concuerdan con los resultados de este estudio ^1^^,^^9^^,^^15^^,^^16^. Aunque en la población estudiada predominó el sexo femenino, debido a factores sociodemográficos y la exposición a riesgos (como el humo de leña), los datos respaldan consistentemente la idea de que la edad y el sexo masculino son determinantes para prever la mortalidad en personas con enfermedades pulmonares ^14^.

Los fármacos anticolinérgicos desempeñan un papel fundamental en el control de la obstrucción del flujo aéreo, una característica esencial de los pacientes con EPOC ^15^. Los beneficios clínicos de estos tratamientos, en términos de eficacia y seguridad, se han corroborado en ensayos clínicos controlados aleatorizados ^15^^-^^19^. De igual manera, se reconoce que la vacunación es una medida que disminuye el riesgo de exacerbaciones y que puede influir de manera favorable en los sujetos con EPOC ^3^^,^^4^. El hecho de que los resultados muestren una mayor frecuencia de terapia inhalada anticolinérgica y de vacunación en pacientes fallecidos puede estar relacionado con un mayor número de intervenciones terapéuticas en pacientes con condición más grave.

La malnutrición, en especial aquella que deriva en sobrepeso y obesidad, tiene resultados clínicos variables con desenlaces como la muerte ^20^^,^^21^. DeLapp y colaboradores analizaron desenlaces clínicos -como la duración de la estancia hospitalaria debida a una exacerbación- y la mortalidad en 301 pacientes con diagnóstico de EPOC, de los cuales el 41 % (122 / 301) era obeso ^21^. Los autores mencionaron que los pacientes obesos tuvieron una reducción significativa de las probabilidades de morir, pero tasas más altas de comorbilidades. En este estudio se encontró un mayor peso e índice de masa corporal en los pacientes con desenlaces fatales, junto con una mayor frecuencia de comorbilidades. Además, las enfermedades de tejido conjuntivo y los procesos tumorales presentaron mayores tasas de mortalidad, sin embargo, el número de sujetos con estas entidades fue muy bajo por lo que esta asociación debe analizarse con cuidado.

El presente estudio está limitado por su diseño retrospectivo, ya que la calidad de la información está restringida a lo registrado en la historia clínica. No obstante, la información fue recolectada por personal médico entrenado y con conocimiento del protocolo. Se necesitan estudios poblacionales con mayor número de sujetos e inclusión de otras variables clínicas para evaluar otros factores relacionados con la mortalidad de los pacientes con EPOC.

La edad y el sexo masculino son variables asociadas con una mayor mortalidad de pacientes con EPOC a 12 meses de seguimiento; los años de exposición al humo de leña, el peso, la insuficiencia cardiaca crónica, la enfermedad cerebrovascular y renal crónica también podrían estar asociados con desenlaces desfavorables, sin embargo, en el presente trabajo no tuvieron significancia estadística.
